# 4-Methoxy­benzene­carbothio­amide

**DOI:** 10.1107/S1600536810015825

**Published:** 2010-05-08

**Authors:** Saqib Ali, Shahid Hameed, Ahmad Luqman, Tashfeen Akhtar, Masood Parvez

**Affiliations:** a Department of Chemistry, Quaid-i-Azam University, Islamabad 45320, Pakistan; b Department of Chemistry, The University of Calgary, 2500 University Drive NW, Calgary, Alberta, Canada T2N 1N4

## Abstract

The asymmetric unit of the title compound, C_8_H_9_NOS, contains two independent mol­ecules with the meth­oxy groups oriented in opposite conformations. The mean planes of the carbothio­amide groups are tilted by 7.88 (15) and 11.16 (9)° from the mean planes of the benzene rings. In the crystal, the mol­ecules form dimers *via* intermolecular N—H⋯S inter­molecular hydrogen bonds, resulting in eight-membered rings of *R*_2_^2^(8) graph-set motif. The dimers are further linked by C—H⋯O hydrogen bonds into chains along the *c* axis. Adjacent chains inter­act through inter­molecular N—H⋯S hydrogen bonds, generating eight-membered rings of *R*_4_^2^(8) graph-set motif.

## Related literature

For the synthesis, biological activity and applications of thio­amides, see: Zahid *et al.* (2009 ▶); Klimesova *et al.* (1999 ▶); Jagodzinski (2003 ▶); Lebana *et al.* (2008 ▶). For related structures, see: Khan *et al.* (2009*a* ▶,*b* ▶,*c* ▶); Jian *et al.* (2006 ▶). For graph-set notation, see: Bernstein *et al.* (1994 ▶).
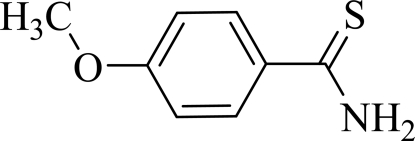


## Experimental

### 

#### Crystal data

C_8_H_9_NOS
*M* *_r_* = 167.22
Orthorhombic, 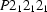

*a* = 5.6545 (2) Å
*b* = 7.3966 (2) Å
*c* = 38.7497 (13) Å
*V* = 1620.67 (9) Å^3^
*Z* = 8
Mo *K*α radiation
μ = 0.34 mm^−1^
*T* = 173 K
0.12 × 0.10 × 0.08 mm

#### Data collection

Nonius KappaCCD diffractometer
Absorption correction: multi-scan (*SORTAV*; Blessing, 1997 ▶) *T*_min_ = 0.961, *T*_max_ = 0.974
6598 measured reflections
3656 independent reflections
3500 reflections with *I* > 2σ(*I*)
*R*_int_ = 0.025

#### Refinement

*R*[*F* ^2^ > 2σ(*F* ^2^)] = 0.033
*wR*(*F* ^2^) = 0.078
*S* = 1.09
3656 reflections
213 parameters
H atoms treated by a mixture of independent and constrained refinement
Δρ_max_ = 0.22 e Å^−3^
Δρ_min_ = −0.27 e Å^−3^
Absolute structure: Flack (1983 ▶), 1469 Friedel pairs
Flack parameter: 0.03 (7)

### 

Data collection: *COLLECT* (Hooft, 1998 ▶); cell refinement: *DENZO* (Otwinowski & Minor, 1997 ▶); data reduction: *SCALEPACK* (Otwinowski & Minor, 1997 ▶); program(s) used to solve structure: *SHELXS97* (Sheldrick, 2008 ▶); program(s) used to refine structure: *SHELXL97* (Sheldrick, 2008 ▶); molecular graphics: *ORTEP-3 for Windows* (Farrugia, 1997 ▶); software used to prepare material for publication: *SHELXL97*.

## Supplementary Material

Crystal structure: contains datablocks global, I. DOI: 10.1107/S1600536810015825/rz2437sup1.cif

Structure factors: contains datablocks I. DOI: 10.1107/S1600536810015825/rz2437Isup2.hkl

Additional supplementary materials:  crystallographic information; 3D view; checkCIF report

## Figures and Tables

**Table 1:** Hydrogen-bond geometry (Å, °)

*D*—H⋯*A*	*D*—H	H⋯*A*	*D*⋯*A*	*D*—H⋯*A*
N1—H1*A*⋯S1^i^	0.85 (3)	2.79 (3)	3.383 (2)	129 (2)
N11—H11*B*⋯S11^ii^	0.88 (3)	2.63 (2)	3.286 (2)	132 (2)
C8—H8*B*⋯O11^iii^	0.98	2.54	3.382 (3)	144
N1—H1*B*⋯S11	0.91 (2)	2.47 (3)	3.368 (2)	168 (2)
N11—H11*A*⋯S1	0.87 (2)	2.57 (2)	3.420 (2)	165 (2)
C2—H2⋯S1	0.95	2.69	3.100 (2)	107
C12—H12⋯S11	0.95	2.70	3.103 (2)	106

Symmetry codes: (i) 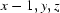; (ii) 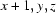; (iii) 
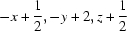
.

**Fig. 1.**
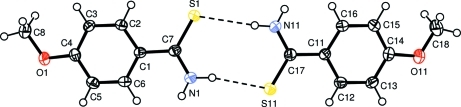
The molecular structure of the title compound plotted with displacement ellipsoids at the 50% probability level (Farrugia, 1997). Intermolecular hydrogen bonds have been presented by dashed lines.

**Fig. 2.**
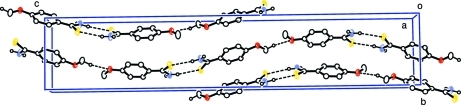
A unit cell showing intermolecular hydrogen bonds by dashed lines resulting in chains of molecules along the c-axis. The H-atoms not involved in H-bonds have been excluded for clarity.

**Fig. 3.**
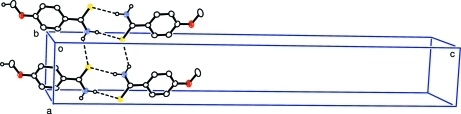
A part of the unit cell showing intermolecular hydrogen bonds of the N—H···S type resulting in eight membered rings generating R22(8) and R42(8) graph-set motifs. The H-atoms not involved in H-bonds have been excluded for clarity.

## supplementary crystallographic information

### Comment

Thioamides exhibit a wide range of applications, not only as synthetic intermediates in the synthesis of a variety of heterocyclic compounds (Zahid *et al.*, 2009), but also numerous biological activities have been associated with them (Jagodzinski, 2003; Klimesova *et al.*, 1999). Moreover, thioamides are important ligands in the field of coordination chemistry (Lebana *et al.*, 2008). In continuation to our work on thioamides (Khan *et al.*, 2009*a*; 2009*b*; 2009*c*), we have synthesized 4-methoxybenzothioamide. In this article we report the crystal structure of the title compound.

The title structure contains two conformational isomers, molecule A and B, containing atoms S1 and S11, respectively, in an asymmetric unit with methoxy groups oriented in opposite conformations (Fig. 1). The mean-planes of the carbothioamide groups (S/N/C) are tilted by 7.88 (15) and 11.16 (9)° from the mean-planes of the phenyl rings in molecules A and B, respectively. The dihedral angle between the mean-planes of the phenyl rings of the two molecules is 58.57 (4)°. The molecules A and B form dimers *via* N—H···S type intermolecular hydrogen bonds resulting in eight membered rings in R_2_^2^(8) motif (Bernstein *et al.*, 1994). The dimers are further linked by C8—H8B···O11 hydrogen bonds into chains along the *c*-axis (Fig. 2). The adjacent chains of molecules are held together by N—H···S type intermolecular hydrogen bonds resulting in eight membered rings in R_4_^2^(8) motif (Fig. 3); details of hydrogen bonding geometry have been provided in Table 1.

The bond distances and angles in both molecules agree with the cortresponding bond distances and angles reported in closely related compounds (Khan *et al.*, 2009*a*; 2009*b*; 2009*c*; Jian *et al.*, 2006).

### Experimental

A slurry of magnesium cholride hexahydrate (5.8 mmol) and sodium hydrogen sulphide hydrate (70%, 11.6 mmol) was prepared in dimethylformamide (15 ml). 4-Methoxybenzonitrile (5.8 mmol) was added to the slurry and the reaction mixture was stirred at room temperature for 5 h. The reaction mixture was poured into water (60 ml) and the resulting precipitates were collected by filtration. The product obtained was resuspended in 1 N HCl (30 ml), stirred for another 25 min, the precipitated solid filtered and washed with water. Recrystallization of the product from chloroform afforded the crystals of the title compound suitable for X-ray crystallographic analysis.

### Refinement

Though all the H atoms could be distinguished in the difference Fourier map the H-atoms bonded to C-atoms were included at geometrically idealized positions and refined in riding-model approximation with C—H = 0.95 and 0.98 Å for aryl and methyl H-atoms, respectively. The H-atoms bonded to N atoms were allowed to refine. The *U*_iso_(H) were allowed at 1.2/1.5*U*_eq_(N/C). The final difference map was essentially featurless.

### Crystal data

**Table:**

C_8_H_9_NOS	*F*(000) = 704
*M**_r_* = 167.22	*D*_x_ = 1.371 Mg m^−^^3^
Orthorhombic, *P*2_1_2_1_2_1_	Mo *K*α radiation, λ = 0.71073 Å
Hall symbol: P 2ac 2ab	Cell parameters from 3578 reflections
*a* = 5.6545 (2) Å	θ = 1.0–27.5°
*b* = 7.3966 (2) Å	µ = 0.34 mm^−^^1^
*c* = 38.7497 (13) Å	*T* = 173 K
*V* = 1620.67 (9) Å^3^	Prism, yellow
*Z* = 8	0.12 × 0.10 × 0.08 mm

### Data collection

**Table:**

Nonius KappaCCD diffractometer	3656 independent reflections
Radiation source: fine-focus sealed tube	3500 reflections with *I* > 2σ(*I*)
graphite	*R*_int_ = 0.025
ω and φ scans	θ_max_ = 27.5°, θ_min_ = 2.8°
Absorption correction: multi-scan (*SORTAV*; Blessing, 1997)	*h* = −6→7
*T*_min_ = 0.961, *T*_max_ = 0.974	*k* = −9→9
6598 measured reflections	*l* = −50→49

### Refinement

**Table:**

Refinement on *F*^2^	Secondary atom site location: difference Fourier map
Least-squares matrix: full	Hydrogen site location: difference Fourier map
*R*[*F*^2^ > 2σ(*F*^2^)] = 0.033	H atoms treated by a mixture of independent and constrained refinement
*wR*(*F*^2^) = 0.078	*w* = 1/[σ^2^(*F*_o_^2^) + (0.0226*P*)^2^ + 0.7048*P*] where *P* = (*F*_o_^2^ + 2*F*_c_^2^)/3
*S* = 1.09	(Δ/σ)_max_ = 0.001
3656 reflections	Δρ_max_ = 0.22 e Å^−^^3^
213 parameters	Δρ_min_ = −0.27 e Å^−^^3^
0 restraints	Absolute structure: Flack (1983), 1469 Friedel pairs
Primary atom site location: structure-invariant direct methods	Flack parameter: 0.03 (7)

### Special details

**Table:**

Geometry. All esds (except the esd in the dihedral angle between two l.s. planes) are estimated using the full covariance matrix. The cell esds are taken into account individually in the estimation of esds in distances, angles and torsion angles; correlations between esds in cell parameters are only used when they are defined by crystal symmetry. An approximate (isotropic) treatment of cell esds is used for estimating esds involving l.s. planes.
Refinement. Refinement of *F*^2^ against ALL reflections. The weighted *R*-factor wR and goodness of fit *S* are based on *F*^2^, conventional *R*-factors *R* are based on *F*, with *F* set to zero for negative *F*^2^. The threshold expression of *F*^2^ > σ(*F*^2^) is used only for calculating *R*-factors(gt) etc. and is not relevant to the choice of reflections for refinement. *R*-factors based on *F*^2^ are statistically about twice as large as those based on *F*, and *R*- factors based on ALL data will be even larger.

### Fractional atomic coordinates and isotropic or equivalent isotropic displacement parameters (Å^2^)

**Table:**

	*x*	*y*	*z*	*U*_iso_*/*U*_eq_	
S1	0.54377 (9)	0.80205 (8)	0.411169 (12)	0.03778 (14)	
S11	0.00254 (9)	0.83920 (9)	0.329665 (12)	0.04206 (15)	
O1	0.3345 (2)	1.12722 (19)	0.56954 (3)	0.0314 (3)	
O11	0.2829 (2)	0.8073 (2)	0.16293 (3)	0.0372 (3)	
N1	0.1172 (3)	0.9318 (3)	0.41309 (5)	0.0384 (4)	
H1A	−0.015 (5)	0.958 (3)	0.4224 (6)	0.046*	
H1B	0.109 (4)	0.901 (3)	0.3904 (7)	0.046*	
N11	0.4395 (3)	0.7318 (3)	0.32528 (4)	0.0380 (4)	
H11A	0.445 (4)	0.734 (3)	0.3477 (6)	0.046*	
H11B	0.577 (4)	0.720 (3)	0.3147 (6)	0.046*	
C1	0.3167 (3)	0.9530 (2)	0.46743 (4)	0.0215 (3)	
C2	0.5056 (3)	0.9037 (2)	0.48859 (4)	0.0233 (3)	
H2	0.6295	0.8321	0.4792	0.028*	
C3	0.5173 (3)	0.9561 (2)	0.52284 (4)	0.0245 (3)	
H3	0.6481	0.9211	0.5367	0.029*	
C4	0.3364 (3)	1.0602 (2)	0.53680 (5)	0.0247 (4)	
C5	0.1422 (3)	1.1069 (2)	0.51645 (5)	0.0274 (4)	
H5	0.0164	1.1753	0.5261	0.033*	
C6	0.1329 (3)	1.0540 (2)	0.48245 (5)	0.0250 (4)	
H6	−0.0003	1.0863	0.4688	0.030*	
C7	0.3124 (3)	0.9001 (2)	0.43058 (5)	0.0240 (4)	
C8	0.5271 (4)	1.0780 (3)	0.59156 (5)	0.0432 (5)	
H8A	0.5297	0.9464	0.5945	0.065*	
H8B	0.5073	1.1360	0.6141	0.065*	
H8C	0.6762	1.1178	0.5812	0.065*	
C11	0.2594 (3)	0.7796 (2)	0.26992 (4)	0.0223 (3)	
C12	0.0790 (3)	0.8599 (2)	0.25047 (5)	0.0272 (4)	
H12	−0.0537	0.9112	0.2619	0.033*	
C13	0.0917 (3)	0.8656 (3)	0.21488 (5)	0.0292 (4)	
H13	−0.0319	0.9206	0.2020	0.035*	
C14	0.2848 (3)	0.7913 (3)	0.19784 (5)	0.0274 (4)	
C15	0.4646 (3)	0.7085 (2)	0.21656 (4)	0.0286 (4)	
H15	0.5957	0.6557	0.2050	0.034*	
C16	0.4502 (3)	0.7039 (2)	0.25229 (4)	0.0272 (4)	
H16	0.5734	0.6478	0.2651	0.033*	
C17	0.2475 (3)	0.7799 (2)	0.30819 (5)	0.0256 (4)	
C18	0.4835 (5)	0.7421 (4)	0.14431 (5)	0.0517 (6)	
H18A	0.6267	0.8017	0.1529	0.078*	
H18B	0.4638	0.7688	0.1197	0.078*	
H18C	0.4976	0.6112	0.1476	0.078*	

### Atomic displacement parameters (Å^2^)

**Table:**

	*U*^11^	*U*^22^	*U*^33^	*U*^12^	*U*^13^	*U*^23^
S1	0.0264 (2)	0.0619 (3)	0.0250 (2)	0.0131 (2)	−0.00098 (18)	−0.0060 (2)
S11	0.0233 (2)	0.0758 (4)	0.0271 (2)	0.0073 (3)	0.00048 (19)	−0.0079 (2)
O1	0.0349 (7)	0.0362 (7)	0.0229 (6)	0.0047 (6)	0.0003 (6)	−0.0048 (5)
O11	0.0409 (8)	0.0482 (8)	0.0225 (6)	0.0052 (7)	−0.0012 (6)	0.0014 (6)
N1	0.0257 (8)	0.0610 (12)	0.0286 (9)	0.0113 (8)	−0.0050 (7)	−0.0105 (9)
N11	0.0262 (8)	0.0637 (12)	0.0241 (8)	0.0107 (8)	−0.0027 (7)	−0.0031 (8)
C1	0.0187 (8)	0.0204 (7)	0.0253 (8)	−0.0012 (6)	0.0007 (7)	0.0025 (7)
C2	0.0214 (8)	0.0221 (8)	0.0264 (8)	0.0025 (7)	0.0018 (7)	0.0003 (6)
C3	0.0248 (9)	0.0248 (8)	0.0238 (8)	0.0023 (7)	−0.0044 (7)	0.0016 (6)
C4	0.0275 (9)	0.0230 (8)	0.0235 (8)	−0.0024 (7)	0.0023 (7)	0.0006 (7)
C5	0.0270 (9)	0.0245 (8)	0.0308 (9)	0.0033 (7)	0.0043 (7)	−0.0010 (7)
C6	0.0202 (8)	0.0234 (8)	0.0313 (9)	0.0024 (7)	−0.0003 (7)	0.0008 (7)
C7	0.0203 (8)	0.0246 (8)	0.0272 (9)	−0.0018 (7)	−0.0013 (7)	0.0023 (7)
C8	0.0461 (12)	0.0573 (13)	0.0262 (9)	0.0095 (12)	−0.0060 (10)	−0.0073 (9)
C11	0.0209 (8)	0.0222 (8)	0.0238 (8)	−0.0023 (7)	−0.0013 (6)	0.0003 (7)
C12	0.0220 (8)	0.0297 (9)	0.0299 (9)	0.0009 (7)	0.0001 (7)	−0.0010 (7)
C13	0.0263 (9)	0.0317 (10)	0.0296 (9)	0.0044 (8)	−0.0062 (7)	0.0000 (8)
C14	0.0327 (9)	0.0262 (9)	0.0234 (8)	−0.0037 (8)	−0.0022 (7)	0.0001 (7)
C15	0.0277 (9)	0.0307 (9)	0.0273 (8)	0.0045 (8)	0.0019 (7)	−0.0024 (7)
C16	0.0252 (9)	0.0291 (9)	0.0273 (8)	0.0051 (8)	−0.0015 (7)	0.0002 (7)
C17	0.0232 (8)	0.0273 (9)	0.0262 (9)	−0.0039 (7)	−0.0007 (7)	−0.0008 (7)
C18	0.0535 (14)	0.0752 (16)	0.0263 (9)	0.0138 (14)	0.0077 (10)	−0.0003 (10)

### Geometric parameters (Å, °)

**Table:**

S1—C7	1.6744 (19)	C5—C6	1.376 (3)
S11—C17	1.6742 (18)	C5—H5	0.9500
O1—C4	1.362 (2)	C6—H6	0.9500
O1—C8	1.431 (2)	C8—H8A	0.9800
O11—C14	1.358 (2)	C8—H8B	0.9800
O11—C18	1.428 (3)	C8—H8C	0.9800
N1—C7	1.316 (2)	C11—C16	1.394 (2)
N1—H1A	0.85 (3)	C11—C12	1.401 (2)
N1—H1B	0.91 (2)	C11—C17	1.484 (2)
N11—C17	1.321 (2)	C12—C13	1.381 (2)
N11—H11A	0.87 (2)	C12—H12	0.9500
N11—H11B	0.88 (3)	C13—C14	1.389 (3)
C1—C2	1.395 (2)	C13—H13	0.9500
C1—C6	1.406 (2)	C14—C15	1.391 (2)
C1—C7	1.481 (2)	C15—C16	1.387 (2)
C2—C3	1.384 (2)	C15—H15	0.9500
C2—H2	0.9500	C16—H16	0.9500
C3—C4	1.390 (2)	C18—H18A	0.9800
C3—H3	0.9500	C18—H18B	0.9800
C4—C5	1.395 (3)	C18—H18C	0.9800
			
C4—O1—C8	117.17 (15)	H8A—C8—H8B	109.5
C14—O11—C18	117.87 (16)	O1—C8—H8C	109.5
C7—N1—H1A	124.0 (16)	H8A—C8—H8C	109.5
C7—N1—H1B	119.9 (16)	H8B—C8—H8C	109.5
H1A—N1—H1B	115 (2)	C16—C11—C12	118.03 (16)
C17—N11—H11A	121.8 (16)	C16—C11—C17	121.70 (16)
C17—N11—H11B	121.3 (15)	C12—C11—C17	120.25 (16)
H11A—N11—H11B	116 (2)	C13—C12—C11	120.82 (17)
C2—C1—C6	117.49 (16)	C13—C12—H12	119.6
C2—C1—C7	120.67 (16)	C11—C12—H12	119.6
C6—C1—C7	121.84 (16)	C12—C13—C14	120.21 (17)
C3—C2—C1	121.81 (16)	C12—C13—H13	119.9
C3—C2—H2	119.1	C14—C13—H13	119.9
C1—C2—H2	119.1	O11—C14—C13	115.64 (16)
C2—C3—C4	119.53 (17)	O11—C14—C15	124.33 (17)
C2—C3—H3	120.2	C13—C14—C15	120.04 (16)
C4—C3—H3	120.2	C16—C15—C14	119.24 (17)
O1—C4—C3	124.76 (17)	C16—C15—H15	120.4
O1—C4—C5	115.45 (16)	C14—C15—H15	120.4
C3—C4—C5	119.77 (16)	C15—C16—C11	121.65 (17)
C6—C5—C4	120.07 (17)	C15—C16—H16	119.2
C6—C5—H5	120.0	C11—C16—H16	119.2
C4—C5—H5	120.0	N11—C17—C11	117.58 (16)
C5—C6—C1	121.29 (17)	N11—C17—S11	120.10 (14)
C5—C6—H6	119.4	C11—C17—S11	122.32 (13)
C1—C6—H6	119.4	O11—C18—H18A	109.5
N1—C7—C1	117.58 (17)	O11—C18—H18B	109.5
N1—C7—S1	120.09 (15)	H18A—C18—H18B	109.5
C1—C7—S1	122.33 (13)	O11—C18—H18C	109.5
O1—C8—H8A	109.5	H18A—C18—H18C	109.5
O1—C8—H8B	109.5	H18B—C18—H18C	109.5
			
C6—C1—C2—C3	−2.0 (3)	C16—C11—C12—C13	−0.7 (3)
C7—C1—C2—C3	178.01 (16)	C17—C11—C12—C13	177.91 (17)
C1—C2—C3—C4	0.2 (3)	C11—C12—C13—C14	0.0 (3)
C8—O1—C4—C3	−3.6 (3)	C18—O11—C14—C13	176.58 (19)
C8—O1—C4—C5	178.21 (18)	C18—O11—C14—C15	−3.0 (3)
C2—C3—C4—O1	−176.50 (17)	C12—C13—C14—O11	−178.65 (17)
C2—C3—C4—C5	1.6 (3)	C12—C13—C14—C15	0.9 (3)
O1—C4—C5—C6	176.62 (16)	O11—C14—C15—C16	178.48 (18)
C3—C4—C5—C6	−1.7 (3)	C13—C14—C15—C16	−1.0 (3)
C4—C5—C6—C1	−0.1 (3)	C14—C15—C16—C11	0.3 (3)
C2—C1—C6—C5	1.9 (3)	C12—C11—C16—C15	0.6 (3)
C7—C1—C6—C5	−178.06 (17)	C17—C11—C16—C15	−178.02 (17)
C2—C1—C7—N1	172.04 (18)	C16—C11—C17—N11	10.3 (3)
C6—C1—C7—N1	−8.0 (3)	C12—C11—C17—N11	−168.26 (18)
C2—C1—C7—S1	−7.6 (2)	C16—C11—C17—S11	−169.92 (14)
C6—C1—C7—S1	172.40 (14)	C12—C11—C17—S11	11.5 (2)

### Hydrogen-bond geometry (Å, °)

**Table:**

*D*—H···*A*	*D*—H	H···*A*	*D*···*A*	*D*—H···*A*
N1—H1A···S1^i^	0.85 (3)	2.79 (3)	3.383 (2)	129 (2)
N11—H11B···S11^ii^	0.88 (3)	2.63 (2)	3.286 (2)	132 (2)
C8—H8B···O11^iii^	0.98	2.54	3.382 (3)	144
N1—H1B···S11	0.91 (2)	2.47 (3)	3.368 (2)	168 (2)
N11—H11A···S1	0.87 (2)	2.57 (2)	3.420 (2)	165 (2)
C2—H2···S1	0.95	2.69	3.100 (2)	107
C12—H12···S11	0.95	2.70	3.103 (2)	106

Symmetry codes: (i) *x*−1, *y*, *z*; (ii) *x*+1, *y*, *z*; (iii) −*x*+1/2, −*y*+2, *z*+1/2.
